# Do Applicant Reactions to Gamified Cognitive Ability Tests Differ Between High- Versus Low-Stakes Settings?

**DOI:** 10.3390/jintelligence13030033

**Published:** 2025-03-07

**Authors:** Marie L. Ohlms, Klaus G. Melchers

**Affiliations:** 1 Institute of Psychology, Albert-Ludwigs-Universität Freiburg, 79085 Freiburg, Germany; 2 Institute of Psychology and Education, Universität Ulm, 89069 Ulm, Germany

**Keywords:** gamification, cognitive ability test, personnel selection, applicant reactions, game, assessment, applicant perceptions

## Abstract

Although cognitive ability tests are among the best predictors of job and training performance, their acceptance among applicants is limited. However, with the current talent shortage, applicant reactions to assessments have become increasingly important. Gamification is a promising approach for improving reactions to cognitive ability tests. However, it remains unclear how findings from low-stakes studies of gamified assessments generalize to high-stakes settings. In this quasi-experimental study (*N* = 210), we compared reactions to a gamified cognitive test from a low-stakes simulated selection setting with experimental participants and from a high-stakes selection setting with real applicants. Test takers in both settings completed the same gamified cognitive ability test and then rated several applicant reactions variables. We found a clear effect of the test setting with real applicants showing more positive reactions to the gamified test concerning perceived fairness, test motivation, organizational attractiveness, behavioral intentions, organizational image, clarity of work activity, and enjoyment compared to participants in the low-stakes setting, whereas there were no differences for perceived job-relatedness and opportunity to perform. These findings highlight the influence of test setting on applicant reactions and underscore the importance of examining environmental factors for research on the effects of gamification in cognitive ability testing.

## 1. Introduction

Traditional cognitive ability tests are well established in personnel selection and assessment, as they are among the most valid predictors of job performance (Sackett et al. 2022; Salgado et al. 2003) as well as for training performance (Hülsheger et al. 2007; Schmidt and Hunter 1998) and educational achievement (Kuncel and Hezlett 2007). However, despite their predictive validity, traditional cognitive ability tests have the disadvantage that they are only moderately well accepted by applicants who may perceive them as unrelated to the job or as too stressful (Hausknecht et al. 2004). This is problematic, firstly because negative applicant reactions can damage the attractiveness of organizations and can reduce the likelihood of job offers being accepted (Hausknecht et al. 2004). Secondly, in the current context of global talent shortages, organizations cannot afford to deter qualified applicants by using negatively perceived selection methods (Vaiman et al. 2021).

To address this challenge, researchers and practitioners have sought to assess applicants’ cognitive ability in ways that preserve predictive validity while enhancing applicant perceptions. One promising approach is the use of gamification of cognitive ability tests (i.e., the integration of game elements such as storylines, points, or leaderboards into assessment contexts, see Landers and Sanchez 2022—or of game-based assessments, i.e., actual games, to measure cognitive abilities, e.g., Bipp et al. (2024)) For example, embedding a traditional non-gamified cognitive ability test into a storyline in which test takers must fight off aliens would transform the test into a gamified version (see Ohlms et al. 2025, for an example). Gamified assessments aim to enhance the candidate experience and improve applicant reactions without sacrificing test validity (Bhatia and Ryan 2018; Woods et al. 2020).

Preliminary research suggests that well-designed gamified assessments tailored to the targeted applicant group can improve perceptions of fairness, enjoyment, and organizational attractiveness (e.g., Hommel et al. 2022; Ohlms et al. 2025). However, much of this evidence comes from hypothetical, laboratory-based studies that do not fully replicate the high-stakes nature of real-world selection settings. In actual job application contexts, applicants may perceive gamified cognitive ability tests differently due to the higher stakes and potential consequences of their test performance, such as receiving a job offer or not (Hausknecht et al. 2004). This raises concerns about the generalizability of low-stakes (laboratory) findings to real-world high-stakes settings and highlights the need to compare applicant reactions from both settings in order to advance theory and practice and to evaluate results of previous research from low-stakes settings.

The present study addresses this research gap by directly comparing reactions to a gamified cognitive ability test in two settings: a high-stakes setting in which real applicants complete the test to gain a job offer and a low-stakes simulated selection scenario in which research participants complete the test. Thus, test takers in both settings completed the same gamified cognitive ability test and subsequently answered questions concerning their reactions to this assessment.

By comparing test taker reactions in a high-stakes and a low-stakes setting, we shed light on the influence of the selection setting on reactions to gamified assessments. Furthermore, we reveal the extent to which results from simulated low-stakes simulated selection settings are comparable to actual high-stakes settings, thus contributing to the theoretical and practical understanding of gamification in cognitive ability testing and its implementation in organizational contexts.

## 2. Theoretical Background

### 2.1. Gamified Cognitive Ability Tests and Applicant Reactions

Research in the field of personnel selection and assessment has long focused on the criterion-related validity and efficiency of selection instruments. Since the late 1980s, however, the applicant perspective and corresponding theories have gained increasing attention (Truxillo and Bauer 2011). This shift can be attributed to the much-described war for talent (Vaiman et al. 2021), as a result of which it is no longer sufficient for a selection instrument to validly predict applicants’ later job performance. Instead, organizations face the challenge of implementing assessments that are both valid and well-accepted by applicants, as negative applicant reactions can deter qualified individuals from actually accepting a job offer (Harold et al. 2016; Konradt et al. 2017). Cognitive ability tests are particularly affected in this regard because although they are widely used due to their strong criterion-related validity (Sackett et al. 2022), they are only moderately well accepted by applicants compared to other, more costly selection instruments, such as work samples or interviews (Anderson et al. 2010; Hausknecht et al. 2004). This has led to efforts to find ways to improve applicant reactions to such tests. Two approaches that have been used in this regard for quite some time are providing explanations about the content, relevance, or validity of cognitive ability tests (e.g., Melchers and Körner 2019) or adding contextualization (i.e., using a work-related context for the abstract cognitive ability test items that might otherwise seem disconnected from actual job tasks, Rynes and Connerley 1993). Another recent approach that is attracting increasing attention is the use of gamification—that is, the integration of game elements into traditional non-gamified selection instruments (Landers and Sanchez 2022).

The theoretical rationale for why gamification may increase applicant reactions to cognitive ability tests can be explained using Gilliland’s (1993) empirically well-supported fairness model. This model proposes 10 rules of procedural fairness (also referred to as perceived procedure characteristics, i.e., job relatedness, opportunity to perform, reconsideration opportunity, consistency of implementation, timely and informative feedback, selection information, honesty toward applicants, recruiter’s interpersonal effectiveness, two-way communication, and propriety of questions) that should affect applicants’ perceptions of the fairness of the selection process. Meta-analytic evidence confirmed that applicants’ perceived fairness of the selection process is crucial because it influences whether applicants actually intend to accept a job offer or to recommend the organizations to others (Hausknecht et al. 2004). Landers and Sanchez (2022) extended Gilliland’s fairness model in the context of gamified assessment to include game elements (e.g., leaderboards, storyline) that are thought to influence perceived procedure characteristics as well as game-related moderators, such as effective game design and prior experience with game elements.

The gamification of traditional cognitive ability tests may be used to enhance three procedural fairness rules outlined in Gilliland’s (1993) fairness model: job-relatedness (i.e., how well a test appears to assess skills or abilities relevant to the job), interpersonal effectiveness (i.e., the degree to which applicants feel respected, valued, and treated warmly throughout the selection process), and selection information (i.e., degree to which applicants are provided with clear explanations about the selection process and its purpose). First, embedding cognitive ability test items in a storyline such as asking applicants to help potential colleagues with their work tasks, may improve the perceived job-relatedness of the test by putting cognitive ability items into context (Ohlms et al. 2025). By framing the items in a work context, it may be easier for applicants to see why they need to take such a cognitive ability test in order to receive a job offer—because they need this ability to do the job. Embedding such a test in a storyline thus goes beyond contextualization (Rynes and Connerley 1993), as all test items are framed within a common narrative. Second, unlike in traditional cognitive ability tests, where applicants typically have no personal interaction with the assessment administrator, gamified assessments offer the potential for direct communication. For example, a gamified assessment can include avatars that answer applicants’ open questions or that communicate appreciation. Third, gamification elements can be used to provide more information about the selection process, the job, or the organization (i.e., concerning Gilliland’s rule of selection information). Including a quiz that provides applicants with information about the job or organizational culture is an example of how Gilliland’s rule of selection information can be targeted through the use of a gamified assessment.

A few recent studies have examined whether gamification can improve applicant reactions compared to traditional cognitive ability tests. However, the results of these studies were not unequivocally positive. Furthermore, independently of whether positive or negative effects were found in previous studies, the effect sizes were usually relatively small.

Concerning positive effects of gamification, Hommel et al. (2022) compared applicant reactions to a gamified version of the Wisconsin Card Sorting Test, which is intended to measure cognitive flexibility, with its traditional version and with a normal cognitive ability test, and they found that the gamified test was perceived more favorably than the traditional version and the cognitive ability test. Similarly, Landers et al. (2022) compared applicant reactions to a cognitive GBA consisting of several puzzle-like mini-games to a paper–pencil cognitive ability tests and found more positive applicant reactions to their GBA. Furthermore, another study by Ohlms et al. (2024a), found that a gamified cognitive ability test was rated more positively than its traditional non-gamified version in terms of opportunity to perform and clarity of work activity, whereas no differences were found between the two tests regarding job-relatedness, procedural fairness, organizational attractiveness, and enjoyment.

In contrast to potential positive effects of gamification, Ohlms et al. (2024b) found more negative reactions to their Minecraft-based GBA measuring fluid intelligence compared to a paper–pencil test measuring the same cognitive abilities. However, in another study, Ohlms et al. (2025) found that differences in the game design elements can make marked differences for perceptions of a test. Specifically, they found that embedding a cognitive ability test in a realistic storyline related to actual work tasks (i.e., assisting potential colleagues with their work tasks) led to more favorable reactions across several dimensions, including perceived modernity of the organization, job-relatedness, organizational attractiveness, and clarity of work activity than the same cognitive ability test without such a storyline. Conversely, the same cognitive ability test embedded in a fictional storyline about fighting aliens elicited higher enjoyment and perceptions of organizational modernity than its non-gamified counterpart but was perceived as less fair. These findings suggest a double-edged nature of gamified assessments: While gamification may increase enjoyment and novelty of the assessment, it may inadvertently reduce perceptions of fairness if the storyline deviates considerably from the job context (Ohlms et al. 2025).

### 2.2. Applicant Reactions in High-Stakes Versus Low-Stakes Settings

Although there is a large body of research on applicant reactions, the majority of this research has been derived from low-stakes settings, where participants were not real applicants but were instructed to imagine that they were applying for a job. Thus, in a meta-analysis by Hausknecht et al. (2004), 61.6% of applicant reaction studies were conducted in low-stakes contexts, while only 38.4% involved actual job applicants. This difference was even larger in a meta-analysis by Truxillo et al. (2009) on the effects of explanations on applicant reactions, in which less than 20% of the effect sizes were from actual selection settings.

Studies examining applicant reactions under low-stakes conditions (e.g., in a simulated setting) may have the advantage that they offer greater potential for experimental control than high-stakes selection scenarios. However, the generalizability of these findings to high-stakes settings remains uncertain. While low-stakes experimental studies provide high internal validity their external validity remains questionable because the stakes are fundamentally different for actual job applicants (Aguinis and Bradley 2014). For individuals who are actually applying for a job, the outcome of the selection process can have far-reaching consequences with considerable impact on their lives. Accordingly, it seems likely that selection tests potentially elicit stronger emotional investment and increased motivation. Conversely, research participants in simulated selection settings typically do not face meaningful consequences of their performance, potentially resulting in lower emotional engagement. Moreover, vignette studies, which require participants to imagine their reactions to an assessment, may pose an even greater challenge to external validity. This is because people often have difficulty accurately predicting their future reactions, leading them to misestimate the intensity and duration of their reactions to hypothetical scenarios (Dong et al. 2024; Wilson and Gilbert 2005). This potential discrepancy between reactions to gamified cognitive ability tests in high-stakes versus low-stakes settings underscores the importance of comparing reactions in both settings to better understand how results from studies conducted in low-stakes settings can be generalized to high-stakes settings.

Meta-analytic evidence supports the notion that applicant reactions differ between high-stakes and low-stakes settings, although no clear trend has emerged as to which type of setting tends to elicit more positive or negative reactions (Hausknecht et al. 2004). Notably, however, these results refer to selection instruments in general but not specifically to cognitive ability tests. Furthermore, to the best of our knowledge, no primary study has directly compared applicant reactions to the same cognitive ability test in high-stakes and low-stakes settings. Moreover, in the context of gamified cognitive ability tests, we are aware of no study that has assessed applicant reactions in a high-stakes context nor any study that has compared reactions in low-stakes and high-stakes settings. This gap in the literature highlights the need for studies that compare applicant reactions to gamified assessments in both settings in order to provide more robust and actionable insights for theory and practice and to better understand to which degree results from one setting generalize to the other setting.

In the current study, we aimed to address this research gap and to answer the question of whether reactions to a gamified cognitive ability test assessed in a low-stakes setting are comparable, and thus generalizable, to those from a real application (i.e., high-stakes) setting.

## 3. Materials and Methods

We used G*Power Version 3.1.9.2 (Faul et al. 2007) and conducted an a priori power analysis to determine the sample size required to test potential differences between the two settings with a power of 0.80. This power analysis revealed a required sample size of *N* = 188 (*n* = 94 per sample) for a one-way multivariate analysis of variances (MANOVA) with seven dependent variables, assuming for a small to medium effect (*f*^2^ = .08).

We used a quasi-experimental design and collected data from two different samples: a high-stakes sample and a low-stakes sample. As explained below, the high-stakes sample consisted of actual job applicants who completed a gamified cognitive ability as part of the usual selection process of an insurance company, and the low-stakes sample consisted of voluntary research participants who had to imagine they were applying for a job and who completed the same test for research purposes.

### 3.1. High-Stakes Sample

#### Sample and Procedure

Applicants who applied for an apprenticeship or a dual study program at a well-known German insurance company and who met the company’s job criteria based on the screening of their application documents were invited to take a gamified cognitive ability test, which was a mandatory part of the company’s selection process. On the last page of the gamified cognitive ability test, all the applicants were asked to voluntarily answer a questionnaire about their reactions to the test they had just completed. Thus, the high-stakes sample only consisted of individuals who had applied for the apprenticeship or the dual study program and who decided to answer our questionnaire.

A total of 104 applicants (48.08% male, 50.96% female, and 0.96% diverse) completed the applicant reaction questionnaire after providing their informed consent. Another four applicants had taken the gamified test and answered our questionnaire but did not consent to having their data analyzed. Applicants’ mean age was 19.32 years (*SD* = 3.71), ranging from 15 to 37 years.

### 3.2. Low-Stakes Sample

#### Sample and Procedure

As the gamified cognitive ability test used in the study was designed to select apprentices and dual students, we aimed to recruit relatively young samples representative of the assessment’s target population. Participants were recruited through email distribution lists from various high schools and universities. Similar to other studies from simulated selection settings (e.g., Law et al. 2016; Oostrom et al. 2019), participants were told that the two participants with best results in the gamified cognitive ability test would receive a cash prize of EUR 50. This was done to motivate participants to participate in the study and to do their best. In addition, students enrolled at the first author’s university could receive course credit for their participation.

A total of 108 participants voluntarily completed the study. They were not applicants but were recruited as research participants for a study on perceptions of web-based selection tests and only completed the test and the subsequent questionnaire for research purposes (data from this sample were also used as part of another study, the results of which were published in an article by Anonymous et al.). Thus, the low-stakes sample consisted of participants for whom test performance was not related to any consequences beyond the chance to win the cash prize.

Prior to data analysis, two participants were excluded because the time needed to complete the questionnaire indicated that they were not completing it conscientiously (i.e., they needed less than 190 s), resulting in a final sample of *N* = 106 participants. Their mean age was 22.23 years (*SD* = 4.51), ranging from 18 to 59 years. 29.25% self-identified as male and 70.75% as female.

Participants in the low-stakes sample were instructed to imagine that they had applied for an apprenticeship-integrated degree program at a well-known German insurance company (the same one that participants in the high-stakes sample had actually applied for) and were now invited to take an online assessment as part of the application process. This scenario was chosen to best reflect the actual target group of the assessment, as these tests were originally designed to select students and apprentices for this company. Then, after obtaining informed consent, participants completed the gamified cognitive ability test, and immediately thereafter, they responded to a questionnaire measuring various applicant reaction variables.

### 3.3. Measures

#### 3.3.1. Gamified Cognitive Ability Test

**Figure 1:**
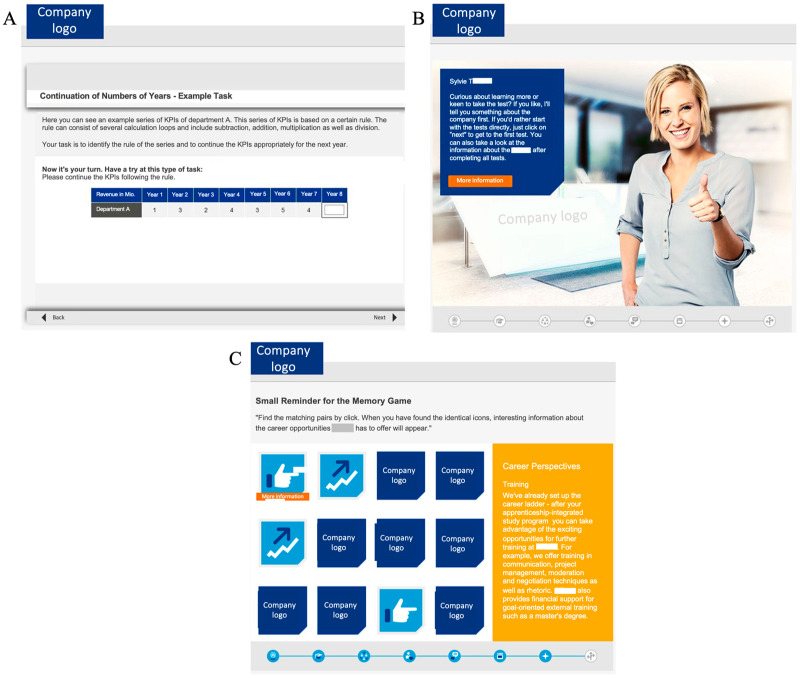
Examples of the gamified test: Example item of the actual cognitive ability test included in the gamified test (Panel **A**), example of the avatars and storyline (Panel **B**), information game (Panel **C**). Note. The actual test was presented to the participants in German. All information about the company has been anonymized. Adapted from CYQUEST GmbH (2017). Copyright 2025 by CYQUEST GmbH. Adapted with permission.

The gamified cognitive ability test utilized in this study was a computer-based cognitive ability test designed for and used by a large German insurance company to pre-select applicants for their apprenticeship and dual study programs. The test consisted of six subtests to measure fluid intelligence and concentration (see Figure 1, Panel A, for an example item).

The gamified cognitive ability test included several game elements in addition to the actual test items. First, the actual test items were embedded in a storyline that was directly related to the insurance company and to relevant work tasks in order to provide a kind of preview of the daily work routine. Specifically, test takers were instructed to imagine that they were going through a trial day at the organization during which they assisted fictional colleagues with their work tasks. These tasks represented the cognitive ability test items, while the storyline emphasized the practical application of these cognitive abilities in the workplace. Second, the avatars represented fictional colleagues who guided the test takers through the test (see Figure 1, Panel B). These fictional colleagues asked the test takers to assist them with various work tasks (i.e., the individual subtests) and provided test takers with additional information about the company and the job. In addition, the test included optional information games designed to present organizational insights in an engaging way: Participants could obtain information or fun facts about the organization by clicking on hidden objects in a search picture, they could complete a quiz game and answer questions about the company, or they could play a memory game in which they could obtain information by revealing two matching pictures (see Figure 1, Panel C). Finally, after completing all subtests, participants had access to an infotainment section where they could revisit the informational games, explore additional details about the company, and gain further impressions. The entire gamified test took approximately 55 min to complete.

#### 3.3.2. Applicant Reactions

All scales and the respective sources are shown in Appendix A. All items were rated on 5-point rating scales ranging from 1 = *strongly disagree* to 5 = *strongly agree*. Measures for which no German version was available were translated into German for the present study (i.e., test motivation).

We used three subscales from the Selection Procedural Justice Scale (Bauer et al. 2001) in the German translation used by Basch et al. (2021) to measure procedural fairness (α = .83), job-relatedness (α = .80), and opportunity to perform (α = .85). Test motivation was assessed with five items by Arvey et al. (1990). To capture organizational attractiveness (α = .94) and behavioral intention (α = .93), five items each from Highhouse et al. (2003) were used in the German translation by Ohlms et al. (2024c). To assess clarity of work activity, we used three items from Ohlms et al. (2024a). Organizational image was measured with three items from Nguyen and Leblanc (2001) in their German translation by Ohlms et al. (2024a). Enjoyment of the test (α = .94) was measured with three items from Wilde et al. (2009).

We conducted a confirmatory factor analysis (CFA) with nine correlated factors to test whether the different reaction variables from the survey indeed represented separable constructs. We used Hu and Bentler’s (1999) criteria to assess model fit (CFI > .96, SRMR < .08, RMSEA < .06) and interpreted the absolute and relative fit indices considering constraints outlined by Marsch et al. (2009). To do so, we used the *lavaan* package in R (Version 4.3.1, Rosseel 2012).

The CFA revealed adequate fit, χ^2^(459) = 839.26, *p* < .001, CFI = .93, SRMR = .05, RMSEA = .06. The loadings ranged from 0.655 to 0.953 with a mean of 0.818. In contrast, a single-factor model had a poor fit, χ^2^(495) = 2667.79, *p* < .001, CFI = .61, SRMR = .15, RMSEA = .15, and fitted significantly worse than the 9-factor model, Δχ^2^(36) = 1828.50, *p* < .001. Owing to a rather high correlation between organizational attractiveness and behavioral intentions (*r* = .93, *p* < .001, *SE* = .009; the average correlation between the other scales was *r* = .39), we further tested a model with eight correlated factors that defined organizational attractiveness and behavioral intentions as one factor, χ^2^(467) = 857.06, *p* < .001; CFI = .93, SRMR = .05, RMSEA = .06. However, this eight-factor model fitted significantly worse than the 9-factor model, Δχ^2^(8) = 17.80, *p* = .02. Consequently, we used the nine applicant reaction variables for the subsequent analyses.

## 4. Results

Means, standard deviations, intercorrelations, and reliabilities for the study variables are presented in Table 1. Furthermore, we assessed potential differences between participants in the high- and low-stakes samples and found that participants in the high- stakes sample were younger, *t*(208) = 5.10, *p* < .001, and included a higher percentage of females, χ^2^(1) = 7.40, *p* = .007, compared to the low-stakes samples. We therefore considered age and gender as covariates in all subsequent analyses.

Concerning the question of whether reactions to a gamified cognitive ability test in a high-stakes selection setting are comparable to those in a low-stakes simulated selection setting, the means of all applicant reaction variables were higher in the high-stakes sample than in the low-stakes sample. Furthermore, for several of the variables, the differences in scale means were larger than a full scale-point on the 5-point rating scale (see Table 2).

To formally test the obtained differences, we conducted three one-way MAN(C)OVAs with the nine applicant reaction variables as dependent variables and the sample (high- vs. low-stakes) as the independent variable, categorizing the dependent variables according to Gilliland’s (1993) justice model. Specifically, one MAN(C)OVA included the procedural justice variables (job-relatedness, opportunity, procedural fairness), another examined the outcomes during testing (test motivation, enjoyment), and the third assessed post-testing outcomes (organizational attractiveness, behavioral intentions, organizational image, and clarity of work activity). Including gender and age as covariates did not change any of the results meaningfully, we decided to report the results in Table 2 without including these variables as covariates. 

The MANOVA with job-relatedness, opportunity, and procedural fairness as dependent variables revealed a small and significant multivariate effect, Wilks’ λ = 0.95, *F*(3, 206) = 3.27, *p* = .02, η^2^*_p_* = .05 (when age and gender were included as covariates, Wilks’ λ was 0.95, *F*(3, 204) = 3.52, *p* = .02, η^2^*_p_* = .05). The MANOVA with test motivation and enjoyment as well as the MANOVA with organizational attractiveness, behavioral intentions, organizational image, and clarity of work activity both revealed large and significant multivariate effects, both Wilks’ λs < 0.73, both *Fs* > 38.99, both *ps* < .001, both η^2^*_p_s* > .26 (when age and gender were included as covariates, both Wilks’ λs < 0.81, both *Fs* > 25.42, both *ps* < .001, both η^2^*_p_s* > .27). Furthermore, subsequent separate one-way ANOVAs revealed large effects for test motivation, organizational attractiveness, behavioral intentions, image, and clarity of work activity (*F*s > 67.78, all *p*s < .001, all *d*s > 1.13), a medium-sized effect for enjoyment (*F*(1, 208) = 11.08, *p* < .001, *d* = .46), and a small effect for procedural fairness (*F*(1, 208) = 7.84, *p* = .006, *d* = .39). However, there were no significant differences for job-relatedness and opportunity to perform, both *F*s < 3.23, both *p*s > .07. Thus, the reactions to the gamified cognitive ability test of real applicants in a high-stakes setting were generally more positive than those of participants in a low-stakes setting but the effects were weaker for the fairness perceptions than for the other applicant reaction variables.

## 5. Discussion

Our results showed more positive reactions in the high-stakes setting than in the low-stakes setting for perceived fairness, test motivation, organizational attractiveness, behavioral intentions, clarity of work activity, organizational image, and enjoyment. Furthermore, most of these differences were rather large and thus much stronger than those commonly observed in many other studies in the field of work and organizational psychology (e.g., Bosco et al. 2015). In contrast, ratings of perceived job-relatedness and opportunity to perform were only descriptively—but not significantly—more positive in the high-stakes setting than in the low-stakes setting. Taken together, these findings suggest that the setting in which a cognitive ability test is administered influences how test takers perceive and evaluate it and that gamification does not affect all applicant reaction variables equally. As we discuss below, our findings have important implications for both research and practice.

### 5.1. Theoretcial and Practical Implications

While applicant reactions have been widely studied, research has predominantly used low-stakes contexts such as hypothetical job applications (Hausknecht et al. 2004; Truxillo et al. 2009). However, for many factors that might affect applicant reaction variables, the external validity is unclear, meaning that it is not sufficiently clear whether we can generalize findings from low-stakes settings to high-stakes settings with real applicants.

In line with previous meta-analytic evidence, our results suggest that the generalizability of applicant reactions from low-stakes settings to high-stakes contexts is limited (Hausknecht et al. 2004; Truxillo et al. 2009). This finding supports Hausknecht et al.’s (2004) applicant reaction model by confirming that the selection setting (low-stakes vs. high-stakes) influences applicant reactions as well as attitudes toward the organization, such as organizational attractiveness and behavioral intentions. Thus, although research conducted in simulated selection contexts often uses incentives (e.g., a cash prize for the top performers such as in the low-stakes condition of the present study or in previous selection simulation studies such as the ones by Law et al. 2016, or by Oostrom et al. 2019) to motivate participants to do their best in order to simulate a selection context, this may not result in the same motivation and reactions that real applicants experience when their careers are on the line. Accordingly, findings from low-stakes simulated selection settings only have limited external validity. Furthermore, because test perceptions and test motivation are related to test takers’ actual performance (Hausknecht et al. 2004), our results suggest that research on influencing factors on test performance (and possibly also on validity) may also only be comparable to a certain extent. However, we are not aware of evidence for qualitative differences between low-stakes and high-stakes settings (i.e., where an influencing factor has positive effects in one setting but negative effects in the other setting) settings. Thus, concerning the external validity of findings from low-stakes settings, our results suggest that effect sizes might not generalize to high-stakes settings—that is, that external validity might be limited in a quantitative manner but not in a qualitative manner.

For organizations, our findings suggest that gamification may indeed be a useful means to improve applicant reactions to cognitive ability tests. Thus, in addition to using explanations (e.g., Truxillo et al. 2009; Melchers and Körner 2019) or contextualized items (e.g., Rynes and Connerley 1993), gamification may positively affect applicant reactions to cognitive ability tests. However, our finding that research on test taker reactions to gamified (or game-based, e.g., Landers et al. 2022) cognitive ability tests might underestimate the reactions of actual applicants stresses the importance of carefully thinking about the specific game elements that should be used. Previous research, for example, found that some game elements might have divergent effects depending on the design choices (e.g., the use of a realistic storyline led to more favorable applicant reactions to a gamified cognitive ability test than the use of a fictional storyline about fighting of aliens in a study by Ohlms et al. 2025, even though both storylines elicited higher enjoyment and perceptions of organizational modernity than a traditional non-gamified test). However, this research might underestimate effects for real applicants given that it was from a low-stakes setting. Thus, using game elements and design choices that lead to more negative perceptions of a test might also have much stronger effects in a high-stakes setting than in a simulated selection setting. Furthermore, if one’s goal is to determine the acceptability of a particular cognitive ability test used to select applicants, it is advisable to obtain feedback from actual job applicants on their reactions to that test, as results from low-stakes settings may underestimate perceptions of real applicants.

### 5.2. Limitations and Future Research

This study is not without limitations and offers potential avenues for future research. One limitation lies in the diversity of gamified assessments, which can differ considerably in terms of the game elements they incorporate and the construct(s) they aim to measure (Ohlms 2024; Ohlms and Melchers 2025; Ohlms et al. 2025). As a consequence, the generalizability of the reactions observed in the present study to other gamified cognitive ability tests—or to gamified personality assessments—should be approached with caution. Nevertheless, we assume that the general qualitative pattern of differences in test taker reactions between high-stakes and low-stakes settings may generalize to other gamified assessments and cognitive ability tests.

A second limitation relates to the fact that we only compared reactions from a high-stakes setting to a low-stakes setting in which participants actually completed the gamified cognitive ability test. However, we excluded an alternative methodological approach that is often used in research on applicant reactions: vignette studies. In vignette studies, participants are instructed to imagine taking a test and then asked about their hypothetical reactions to such an assessment. Future research could examine whether applicant reactions gathered in a low-stakes vignette study differ even more from those observed in a low-stakes simulated selection scenario in which participants actively complete the assessment. In addition, future research could explore how these reactions compare to those of actual applicants in high-stakes contexts, providing a more comprehensive understanding of the contextual influences on applicant reactions.

A final limitation of the present study is that we could not compare differences in test scores. Given that test motivation is related to actual test performance (Hausknecht et al. 2004), it might well be that the differences in test takers’ motivation observed in the present study are also related to differences in actual test scores. However, as we had no access to test scores from the high-stakes setting, we were not able to compare test scores between the two settings.

## 6. Conclusions

Given that most research on applicant reactions to cognitive ability tests has been conducted in low-stakes simulated selection contexts in which participants only imagine applying for a job, the present study sought to explore whether reactions to a gamified cognitive ability test in such settings generalize to those of actual applicants in high-stakes settings. Our results revealed a general pattern that suggests that applicants in a high-stakes setting show more positive reactions to a gamified cognitive ability test than participants taking the same test in a low-stakes simulated selection scenario. Thus, our findings suggest that effects of gamification are stronger in high-stakes settings and that the generalizability of findings concerning applicant reactions from low-stakes settings to high-stakes contexts should be approached with caution, as contextual factors appear to influence perceptions of gamified cognitive ability tests.

## Figures and Tables

**Table 1:** Descriptive information and correlations of the study variables.

Variable	*M*	*SD*	1		2		3		4		5		6		7		8		9		10		11	
*M*				1.71		22.23		2.79		.267		3.47		4.00		3.31		2.80		2.87		3.33		2.75
*SD*				0.46		4.51		0.90		0.75		0.87		0.74		0.94		1.02		0.98		0.72		0.86
1. Gender ^a^	1.51	0.50			−	.19		.10	−	.02		.15	−	.12	−	.03		.06		.15		.15		.09
2. Age	19.32	3.71		.12			−	.04		.13	−	.09	−	.15	−	.09		.08		.03	−	.04		.03
3. Job-relatedness	2.80	0.98	−	.09	−	.04				.49 ***		.44 ***		.02		.08		.41 ***		.34 ***		.13		.32 ***
4. Opportunity	2.88	0.92	−	.13		.04		.51 ***				.57 ***		.09		.34 ***		.37 ***		.31 **		.33 ***		.31 **
5. Fairness	3.79	0.80	−	.12		.20 *		.45 ***		.60 ***				.00		.26 **		.28 **		.28 **		.27 **		.38 ***
6. Test motivation	4.72	0.38	−	.14	−	.28 **		.05		.04		.06				.47 ***		.20 *		.27 **		.15		.07
7. Enjoyment	3.70	0.77	−	.03		.04		.31 **		.56 ***		.47 ***	−	.02				.21 *		.28 **		.20 *		.17
8. Organizational attractiveness	4.70	0.37		.13	−	.03	−	.07	−	.01		.09		.36 ***		.05				.86 ***		.44 ***		.37 ***
9. Behavioral intentions	4.67	0.38	−	.08	−	.01		.03		.05		.06		.32 ***		.17		.65 ***				.48 ***		.32 ***
10. Organizational image	4.52	0.52	−	.11	−	.10		.06		.19		.13		.38 ***		.19 *		.39 ***		.49 ***				.07
11. Clarity of work activity	3.60	0.62	−	.08		.02		.29 **		.34 **		.20 *		.09		.02	−	.12	−	.02		.12		

*Note. n* = 104 for the high-stakes sample and 106 for the low-stakes sample. Intercorrelations for the high-stakes sample are presented below the diagonal, and intercorrelations for the low-takes sample are presented above the diagonal. Gender is coded as 0 = male, 1 = female. pre = measured before the test, post = measured after the test. ^a^ *n* = 103 for the high-stakes sample (the diverse participant was dropped for all correlations involving gender). * *p* < .05. ** *p* < .01. *** *p* < .001.

**Table 2:** Means, standard deviations, and effect sizes (with 95% confidence intervals) for the applicant reaction variables to the gamified cognitive ability test in the high-stakes and low-stakes settings.

Dependent Variable	High-Stakes *n* = 104		Low-Stakes *n* = 106		Cohen’s *d* (95% CI)
	*M*	*SD*	*M*	*SD*	
Job-relatedness	2.80	0.98	2.79	0.90	0.02 [−0.25, 0.29]
Opportunity to perform	2.88	0.92	2.67	0.75	0.25 [−0.02, 0.52]
Prodecural fairness	3.79	0.80	3.47	0.87	0.39 ** [0.11, 0.66]
Test motivation	4.72	0.38	4.00	0.74	1.22 *** [0.92, 1.51]
Enjoyment	3.70	0.77	3.31	0.94	0.46 ** [0.18, 0.73]
Organizational attractiveness	4.70	0.37	2.80	1.02	2.47 *** [2.11, 2.82]
Behavioral intentions	4.68	0.38	2.87	0.98	2.41 *** [2.05, 2.76]
Organizational image	4.52	0.52	3.33	0.72	1.90 *** [1.57, 2.23]
Clarity of work activity	3.60	0.62	2.75	0.86	1.14 *** [0.84, 1.43]

*Note.* Positive values for Cohen’s *d* indicate higher means in the high-stakes setting. ** *p* < .01. *** *p* < .001.

## Data Availability

The data that support the findings of this study are openly available from OSF at https://osf.io/mjkd9/?view_only=54214fc5f8aa40c18cc2c9736d0fbf06.

## Appendix A

**Table A1:** Items used to measure the applicant reaction variables.

Scale	Items Used in the Current Study	Source
General procedural fairness	I think that this test is a fair way to select people for the apprenticeship integrated study program. I think that the test itself is fair. Overall, the method used was fair.	Bauer et al. (2001)
Job-relatedness	Doing well on this test means a person can do well in the apprenticeship integrated study program. A person who scored well on this test will also do well in the apprenticeship-integrated degree program.	Bauer et al. (2001)
Opportunity to perform	I could really show my skills and abilities through this test. This test allowed me to show what my job skills are. This test gives applicants the opportunity to show what they can really do. I was able to show what I can do on this test.	Bauer et al. (2001)
Test motivation	I wanted to do well on this test or tests. I tried my best on this test or tests. While taking this test or tests, I concentrated and tried to do well. I want to be among the top scorers on this test. I just didn’t care how I did on this test or tests. ^a^	Arvey et al. (1990)
Organizational attractiveness	For me, this company would be a good place to work. I would not be interested in this company except as a last resort. ^a^ This company is attractive to me as a place for employment. I am interested in learning more about this company. A job at this company is very appealing to me.	Highhouse et al. (2003)
Behavioral Intentions	Iwould accept a job offer from this company. I would make this company one of my first choices as an employer. If this company invited me for another job interview after this procedure, I would go. I would exert a great deal of effort to work for this company. I would recommend this company to a friend looking for a job.	Highhouse et al. (2003)
Clarity of work activity	I have a clear idea of what it is like to work at this company. I know which work tasks would be expected of me in this job. I have a clear idea of what the daily work routine at this company would be like.	Ohlms et al. (2024a)
Organizational image	I have always had a good impression about [name of the organization]. In my opinion, this [name of the organization] has a good image in the minds of consumers. I believe that this [name of the organization] has a better image than its competitors.	Nguyen and Leblanc (2001)
Enjoyment	I enjoyed this test. I find this test very interesting. I find this test entertaining.	Wilde et al. (2009)

*Note.* Items were presented using a 5-point rating scale (1 = *strongly disagree* to 5 = *strongly agree*). All items were presented in German. ^a^ Items are reversed scored.
